# Chicago Veterinary Society

**Published:** 1898-02

**Authors:** James Henderson

**Affiliations:** Secretary


Secretary.

Toronto, Ontario, January 13, 1898.

### CHICAGO VETERINARY SOCIETY.

The regular meeting was held on December 9th at the Sherman House. The following members responded to the roll-call: Dr. R. G. Walker, President; Dr. Jas. Henderson, Secretary; Dr. G. C. McEvers, Treasurer; Drs. A. C. Worms, J. F. Ryan, R. Gysil, L. Campbell, P. Quitman, E. L. Quitman, J. L. Siegroesser, Jos. Hughes, J. Robertson, B. A. Pierce, O. E. Dyson, J. J. McGrath, C. G. Nelson, F. J. Leith, H. H. Hawley, J. B. Clancy, A. O. Caspar, and Frank Allen.

The Secretary read a letter received by him from the Civil Service Board of Commissioners, reiterating their promise to hold an examination at which the veterinarians practising in the city may compete, with a view to selecting a veterinarian to attend the police horses of the city. They, however, evaded stating a definite date at which it would be held. After some discussion the following motion was made by Dr. E. L. Quitman, and seconded by Dr. McGrath:

That the Secretary be instructed to write to the Civil Service Board of Commissioners, asking them to appoint a time at which they would meet the Legislative Committee with a view to obtaining the board's decision regarding the date of the promised examination for police veterinarian. The motion was carried.

Dr. Hughes, chairman of the special committee appointed at our last meeting to draft a guide which would aid us in distinguishing a sound from an unsound or "serviceably sound" horse, when under professional examination, made the following report: The committee found that this work exceeded in volume the time at their disposal to accomplish it. They, however, made a list of 140 blemishes, defects, and diseases which are often met with at the auction mart, and suggested that these matters be discussed and passed upon by the entire Society at our regular meetings. The following motion was made by Dr. Gysil on the subject: That members be appointed at each meeting to lead the discussion on one or more of the abnormal conditions enumerated upon the list, and that a stenographer be employed to report these discussions, with a view to their publication in the veterinary magazines after being edited by the Committee on Literature and Publication. The motion was seconded by Dr. Worms, and carried by the meeting.

The question was again brought up as to what course the Society should take regarding those of its members who still retained their commissions as Assistant State Veterinarians to the present empirical chief. After a lengthy discussion, a motion to postpone all action in the matter until next meeting was carried.

After further business of a routine nature, the meeting was adjourned.

The regular meeting of the Society was held at the Sherman House, on the evening of January 13, 1898, and the following members were present: President, Dr. R. G. Walker; Secretary, Dr. James Henderson, and Drs. R. Gysil, L. Campbell, P. Quitman, E. L. Quitman, Jos. Hughes, L. A. Merillat, J. Robertson, B. A. Pierce, A. H. Baker, C. A. White, O. E. Dyson, C. G. Nelson, F. J. Leith, C. E. Sayre, A. E. Rishel, J. B. Clancy, A. M. Caspar, D. Kennath, Frank Allen, O. R. Dubia, and W. E. McGrath.

The minutes of last meeeting were approved as read.

The Secretary reported a letter sent by him, as instructed at the last meeting, to the Civil Service Board of Commissioners, asking them to appoint a time of meeting with the Legislative Committee. A reply from the secretary of that board was also read, granting our request.

Report of Legislative Committee: Dr. Hughes, as chairman, gave an account of the meeting above arranged for. The board averred that it had many examinations on hand in which large numbers of men were involved. That in the examination for the purpose of selecting a veterinarian to attend police horses only one position was to be filled, and on that account it would be postponed in favor of the examinations at each of which numerous positions were to be disposed of. The board stated, however, that the examination in which we were interested would be held before its term of office expired; that is, before six months; but further that it might be held any time after two weeks, the date depending upon the manner in which they progressed with the preferred examinations. The board also stated that the examination would, if possible, be conducted by expert veterinarians outside of the city, as it would be unfair to have any member of our Society examining both his fellow-members and other practising veterinarians outside of the Society at the same time. This last arrangement was cheerfully accepted by the committee.

Unfinished business: At our meeting on December 9th, a motion was put to the meeting, and carried, that all action in expelling those of our members who retained their commissions as Assistant State Veterinarians to the present empirical chief should be postponed until this meeting. After considerable discussion, the following motion, made by Dr. Campbell, seconded by Secretary, was carried : That a vote by ballot be taken to decide whether these gentlemen be retained as members of the Society or not. Twenty-one ballots were cast—ten for expulsion and eleven for retention.

Regular programme: Dr. Gysil read a paper on the "Operative Treatment of Quittor."

The Society then entered upon the work of drafting "a guide which would aid us in distinguishing a sound from an unsound or a 'serviceably sound' horse when under professional examination." The following are our reporter's notes upon this effort:

Dr. Robertson: I do not see how a horse with diseased or absent molars could be strictly sound, but I have been lax and have not examined many horses' molars. Cribbing is a bad habit, and may lead to unsoundness, and a cribber should be rejected. Wind-sucking is also a cause for rejection.

Misrepresentation of age is a question for the law to decide. We are not supposed to take the dealer's word for the horse's age. Side-pulling is a habit. Wind-sucking I would consider an unsoundness, and cribbing a habit, from the simple fact that I believe it can be traced to disordered digestion.

Wind-sucking is a very serious question, because it is invariably associated with indigestion. Fifty per cent. of cribbers are wind-suckers, and wind-sucking is unsoundness.

It has always been a question in my mind as to whether a veterinary examiner of a horse for soundness should deviate at all from strictly or strict soundness. If we do not deviate from the point of soundness we pass very few horses, and interfere with the horse trade. If I found a diseased condition that would in any way reduce the value I would mention it. Absent molars is one of these cases. I would pass a horse with such a condition as sound if the cavity was closed with cicatricial tissue, unless pinned down to a point of strict soundness.

A horse with absent molars I would pass. My experience bears me out in this. I believe a veterinary examiner should be more or less liberal in his report. He should avoid as much as possible interfering with the horse business or trade. Protect your client, and when it is for his interest reject. Your opinion must be based upon whether the horse's utility is interfered with or not.

Dr. Merillat: I will include diseased and absent molars as one, and speak of them together. On account of the importance of mastication I would consider it a serious lesion. There is no animal in the order of mammalia or herbivora that needs such an extensive mechanism of mastication as does the horse. His food is dry and hard in texture, and must be thoroughly masticated for his gastric digestion, and such diseased molars must lead to serious disease sooner or later, and again disease of one molar is an indication that others are not too sound. You will find by observation that an animal with a diseased molar, if it occurs during the earlier years of his life, is quite sure to have an early disturbance of his whole dental mechanism, as I believe that diseased teeth are due to faulty development.

Side-pulling is a bane to the man practising dentistry. It is a simple habit to the laymen, and one that the laity believe the veterinarian should easily remedy, whether it is caused by abrasions of the buccal membrane or from sharp teeth of any kind. In the latter instance dentists can be of some avail. Another great cause of side-pulling in my observation is lameness or soreness in one side of the body. Navicular disease is another cause. You will find this chiefly in the animal that does not pull much. He is the worst side-puller, and does not take the line, and keeps going from one side of the street to the other. I firmly believe that Dr. Quitman's central cause can be one of the etiological factors in this condition, viz., lesions of the brain, but I do not believe that softening could be put under that head, but chronic hydrocephalus. Ocular disease is another great cause. An animal that has not good and perfect sight might have it from central origin In these cases it is foolish to try and cure the side-puller by removing and cutting the molar.

Dr. Hughes: Diseased teeth in the upper jaw may be a cause of ulceration—a factor which might cause abscess in the maxillary sinus and nasal gleet. I confess I rarely look in the mouth for diseased teeth; at the same time that does not avert the fact that an absent molar means an unsound horse, and I do not think a man is justified in passing a horse with an absent molar. If you extract a diseased tooth that means that the horse is laid up for several weeks.

I hold that cribbing, as a rule, is associated with wind-sucking. Now comes up the question as to what is wind-sucking. Is it a habit by which the animal grasps a mouthful of air and lets it pass down his œsophagus? No; we do not know anything about it.

Misrepresentation of age is a question to be decided by law.

As to the cause of side-pulling and definition, I must confess I have learned a great deal about it to-night. Dr. Wyman says it is a diseased condition of the brain, and the question of dummy came up, also cerebritis and softening of the brain. Dr. Quitman says a dummy means a softening of the brain. Dr. Wyman has taken the position that softening results from a traumatism, and that with the traumatism there must be infection, irritation, and chronic indigestion unless got by irritation. Cribbing, if only a habit, leads to wind-sucking, and wind-sucking leads to constant bloating and irritation of the digestive apparatus. My opinion is that cribbing is an usoundness, and wind-sucking still more so.

Misrepresentation of age: This is a little hard to define as an unsoundness. If the animal is perfectly sound that could not be called an unsoundness. The animal could be returned as being unfit for use if purchased. Forcing the age by extracting the milk-teeth before they are ready to be shed, is easy to tell, and the rejection of the animal should depend upon his acceptability to the buyer.

Side-pulling: This takes in its scope a hard-mouthed animal. Side-puling is a very disagreeable and dangerous habit, and I claim an animal so affected is unsound. Side-pulling may be due to a habit or from sharp or diseased teeth. It is sometimes a result of nasal gleet and brain-pressure. Sometimes there is no diseased condition at all, and he may become a side-puller by having been driven double, and then single. It is a very dangerous habit, especially in crowded cities, as the animal may be the cause of bringing his owner into court. Hard-mouth comes out in driving. It is usually more or less easy to tell by the cicatrized condition of the mouth.

Dr. Dyson: In my experience it is a habit, and due in a great many instances to over-checking or faulty driving. A good remedy for its cure is a change of driver, liberal use of the whip, and to abandon the high checking of the horse. I think in the majority of cases it is merely a habit.

Dr. A. H. Baker: I do not consider a horse with an absent molar and otherwise sound, as unsound. The mere fact that the corresponding molar extending down into this cavity and into the tissues below as being sufficient excuse for rejection of the animal are insufficient. There is no horse that will not require attention sooner or later. They are all composed of flesh and blood, and put into service will all do wrong. Where there is an absent molar the opposing molar needs excising. A horse with a diseased molar is unsound.

Cribbing *per se* is not unsoundness. It is a habit on which the common law will allow a man to return a horse. It is a habit that certainly is very objectionable, and will lead to defects and disorders which we as examiners have nothing to do with.

Misrepresentation of age is not unsoundness. It is a fraud which the common law would punish. It does not interfere with health. The horse is just as healthy as though the age were not misrepresented. A veterinarian always gives his opinion regardless of misrepresentation. If a horse is nine and is represented as six years old, that should not influence the veterinarian, who should give his opinion regardless of whether the seller is telling a lie or not. Misrepresentation is fraud, pure and simple, and not unsoundness.

Side-pulling due to no pathological lesion is not unsoundness. In the majority of cases it is due to sharp teeth. A dentist will correct it. If the horse is lame and is a side-puller it is unsound.

Dr. Wyman: The doctor said that softening in the brain might lead to this trouble. Softening of the brain is either of traumatic or infectious origin, and that it does take place is a fact. We do find it especially as a complication of those troubles where we have a metastasis that may lead to abscess-formation.

I was so forcibly struck with the statement made of softening of the brain and cerebritis, that I thought at some time or other during my life at college that I might have been negligent in gathering the facts, and for that reason only did I beg your permission to speak on the question which before so suddenly came to an end. Hydrocephalus is a condition where we have the lateral ventricles, the third, and never the fourth, filled with serum. This serum is the result of either a transudation or exudation from the choroid plexuses. It may follow an acute lepto-meningitis or an œdema pure and simple. The condition which we usually find on post-mortem is a serum amounting from 20 to 40 grammes—40 being the utmost given by Degraf. Lepto-meningitis is only too often the result of an infection and softening of the brain; never takes place at all, excepting traumatism is present. The point I wish to make, I put it of traumatic origin, with subsequent infection.

Dr. Quitman: In making an examination for soundness, always remember that a horse is a living monument to its examiner, and for that reason it is important to be extremely careful. It is better to be sure than sorry.

Diseased or absent molars: A diseased molar is unquestionably an unsoundness, because (1) that molar will sooner or later cause trouble in mastication of food, or (2) fistula or necrosis of the lower jaw or nasal gleet of the upper. I unquestionably would reject a horse having a decaying or absent molar.

Absent Molar: We must look at this from two sides. If the horse is being sold for a fair price, and being otherwise sound, I would not reject him. Some say it is pretty harsh, yet we know the cavity will lead to several different results. The opposite tooth will sooner or later grow into that cavity, and if not cut off will bruise the opposing jaw. The animal may be serviceably sound.

Cribbing: This I will take up along with wind-sucking. Wind-sucking usually follows cribbing, and when he gets more accomplished he becomes a wind-sucker. A cribber must have some object on which to press his teeth. A cribber may become a wind-sucker in a very short time, and I have seen a horse graduate in one case in about four days. It is certainly a cause to reject a horse. Its simplest fault is ruining the general appearance of the teeth. It is an unsoundness, or it leads to unsoundness.

I mentioned it was due to an injury, and believe the majority of so-called dummy cases could be traced to traumatic injury. It is a common occurrence to see a horse hit over the head with a broomstick or anything of that kind, and the condition that we find in a dummy very closely resembles the symptoms that follow, and, while I do not doubt the condition that any pressure on the brain will cause these conditions, I claim that softening of the brain can be a cause.

In hydrocephalus due to meningtis some of the primary symptoms are those of either motor or sensory irritation, locomotor ataxia or elevation of the legs; on the other hand, the slightest blood-clot may cause a paresis. Now, if we have this effusion into these ventricles large enough to cause this idiotic condition, how is it we do not have paresis? Is it chronic meningtis that causes that abnormal condition? If there is chronic meningitis, what causes these periods of normality? In early stages of cerebral softening they can be brought about by an extra amount of blood being carried to the parts, and dummies can be caused by considerable effusion into the ventricles of the brain and into the cervical portion of the cord and into the subarachnoidean space.

JAMES HENDERSON,
Secretary.

